# High-Resolution Raman Imaging of >300 Patient-Derived Cells from Nine Different Leukemia Subtypes: A Global Clustering Approach

**DOI:** 10.1021/acs.analchem.4c00787

**Published:** 2024-05-31

**Authors:** Renzo Vanna, Andrea Masella, Manuela Bazzarelli, Paola Ronchi, Aufried Lenferink, Cristina Tresoldi, Carlo Morasso, Marzia Bedoni, Giulio Cerullo, Dario Polli, Fabio Ciceri, Giulia De Poli, Matteo Bregonzio, Cees Otto

**Affiliations:** † Istituto di Fotonica e Nanotecnologie − Consiglio Nazionale delle Ricerche (IFN-CNR), c/o Politecnico di Milano, Milan 20133, Italy; ‡ Datrix S.p.A., Milan 20121, Italy; § IRCCS Ospedale San Raffaele, University Vita-Salute San Raffaele, Milan 20132, Italy; ∥ Medical Cell BioPhysics, Department of Science and Technology, TechMed Center, University of Twente, Enschede, NL 7500 AE, The Netherlands; ⊥ Istituti Clinici Scientifici Maugeri IRCCS, Via Maugeri 4, Pavia 27100, Italy; # IRCCS, Fondazione Don Carlo Gnocchi, Milan 20148, Italy; ∇ Dipartimento di Fisica, Politecnico di Milano, Milan 20133, Italy

## Abstract

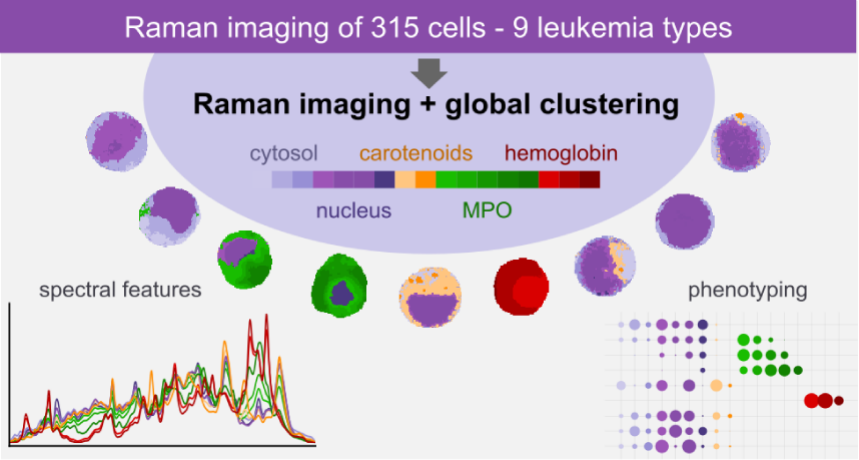

Leukemia comprises a diverse group of bone marrow tumors marked by cell proliferation. Current diagnosis involves identifying leukemia subtypes through visual assessment of blood and bone marrow smears, a subjective and time-consuming method. Our study introduces the characterization of different leukemia subtypes using a global clustering approach of Raman hyperspectral maps of cells. We analyzed bone marrow samples from 19 patients, each presenting one of nine distinct leukemia subtypes, by conducting high spatial resolution Raman imaging on 319 cells, generating over 1.3 million spectra in total. An automated preprocessing pipeline followed by a single-step global clustering approach performed over the entire data set identified relevant cellular components (cytoplasm, nucleus, carotenoids, myeloperoxidase (MPO), and hemoglobin (HB)) enabling the unsupervised creation of high-quality pseudostained images at the single-cell level. Furthermore, this approach provided a semiquantitative analysis of cellular component distribution, and multivariate analysis of clustering results revealed the potential of Raman imaging in leukemia research, highlighting both advantages and challenges associated with global clustering.

Leukemia defines a large group of hematopoietic (blood) tumors characterized by the proliferation of immature cells (called “blasts”) because of alterations occurring at a certain level of hematopoietic maturation in the bone marrow. Myeloid leukemias mainly affect myeloid cells, precursors of blood cell monocytes, granulocytes, megakaryocytes, and erythrocytes; lymphoid leukemias mainly affect lymphoid cells, precursors of lymphocytes.^1−3^ Acute myeloid leukemias (AMLs) and acute lymphoblastic leukemias (ALLs) represent around 40% of all leukemia diagnoses and contain more than 35 subtypes, associated with different prognoses and treatments. Even if the definitive diagnosis may be supported by immunophenotypic and genetic analyses, the visual morphological assessment of bone marrow (BM) and peripheral blood (PB) smears is still a fundamental step.^1−3^ Hematologists have to count and distinguish blasts and other cell subpopulations found in BM or PB smears after manual counting of 500 and/or 200 nucleated cells, respectively.^3^ To this aim, clinicians are usually asked to recognize specific cellular types (e.g., promyelocytes, myeloid precursors, erythroid precursors, and abnormal cells) according to morphological criteria including nuclear-cytoplasmic ratio, cytoplasm granularity (i.e., presence of subcellular organelles), degree of “basophilic” features, etc.

This process is subjective and time-consuming and suffers from intra- and interobserver variability,^4^ especially in the case of cells belonging to the same lineage or with similar maturation stages. Therefore, there is an urgent need to provide clinicians with objective, automated, and accurate approaches for leukemia assessment. In this context, many efforts have been made, including image-based automated methods coupled with machine-learning approaches^5−7^ but they rely on stained samples, implying the same labor-intensive steps and scarce information, based only on morphological criteria.

In contrast, vibrational spectroscopy, such as Raman-based and IR-based approaches, can determine the biomolecular composition of biological samples without the need for staining, thus providing high objectivity and accuracy, and compatibility with automated procedures.^8−10^ By coupling Raman spectroscopy with confocal microscopes, it is also possible to obtain high-quality Raman images at the subcellular level.^11−16^

Various studies reported the use of Raman approaches for the study of leukemia^17−29^ but only part of them included patients’ samples.^18,20−22,24,27,28^ Chan et al. and Managò et al. reported the characterization of leukemia cells derived from ALL patients.^18,22^ Féré et al. and Happillon et al. reported an extended study on smears from 79 chronic lymphocytic leukemia (CLL) patients demonstrating good accuracy in discriminating leukemia cells.^21,24^ However, the above-mentioned studies are based on single-point measurements, most of the time collecting Raman signals from the nucleus. This approach has some advantages, including simplicity and reduced acquisition time, but fails to provide morphological and spatial information and does not provide details about the biomolecular features of cellular cytoplasm, containing key information about cell phenotypes. In addition, the aforementioned Raman studies do not include patients affected by AML, characterized by high biomolecular heterogeneity.

In 2015, we first reported label-free high-resolution Raman imaging of leukemia cells from patients.^20^ 50 cells from 7 patients affected by four AML subtypes (AML M0, M2, M3, and M6) were studied, but only four representative false-color images were produced, with exploratory aims. This was mainly due to the challenge of producing false-color images of several cells by using manually supervised map analysis (i.e., univariate or cluster analysis at the single cell level). Similarly, Leszczenko et al. recently reported Raman imaging of ALL cells from patients, but the imaging approach was mainly used to extract 2/3 main clusters used as an input for further chemometric analysis on averaged spectra.^25^ In both cases, the analytical strategies only partially exploited the spatial information provided by Raman imaging.

Traditionally, Raman imaging data analysis involves univariate, multivariate, or clustering approaches at the level of individual maps (single cells or tissue regions),^10,30^ limiting comprehensive and semiautomatic analysis of the entire data set. Global clustering approaches have been applied to FT-IR hyperspectral maps, aiming to describe the presence and distribution of the same spectral content in different samples or conditions.^31−33^ Nguyen et al. introduced the so-called “joint *k*-means clustering” process for analyzing FT-IR data sets comprising up to 15 tissue images simultaneously.^31^ To date, only one study has applied global clustering to a substantial number of Raman maps.^34^ Authors employed the so-called “common *k*-means analysis” on 118 cells to separate the nucleus and cytoplasm, for subsequent multivariate analysis and not for imaging purposes.

In this study, we conducted a Raman imaging study of leukemia cells derived from 19 patients, each presenting one of nine distinct leukemia subtypes. We collected high spatial resolution (up to 355 nm) maps at the single-cell level, enabling the detection and recognition of specific molecules or subcellular structures that could potentially identify specific subtypes. The final data set consisted of 319 maps and over 1.3 million spectra necessitating the development and optimization of suitable approaches to generate high-quality images and identify distinct morphological and biochemical features across different subtypes. To achieve this, we optimized a single-step global clustering approach that involved simultaneous cluster analysis of all cells in the entire data set. After a semiautomated preprocessing pipeline, the entire data set was thoroughly investigated at the single-pixel level by a single global cluster analysis step, thus automatically providing “virtually stained” cell images and, in parallel, the distribution of relevant biomolecules over the different cells and different subtypes. Furthermore, data emerging from the global cluster analysis were then processed by multivariate analysis with classification aims. Overall, our results underscore the potential of employing a global clustering approach for semiautomated analysis of an extensive data set of Raman maps. Concurrently, we present the most extensive characterization of leukemia subtypes to date using Raman microscopy and Raman imaging methods.

## Experimental Section

### Patients’ Enrolment and Standard Diagnosis

All the patients involved in the study gave written informed consent, as approved by the ethical committee of IRCCS Ospedale San Raffaele and in accordance with the Helsinki guidelines. All the patients have been evaluated through the standard diagnostic workflow according to the most recent WHO guidelines^1−3^ and subjected to morphological, immunophenotypical, cytogenetic, and molecular genetic evaluations. To better focus on morphological features, all the samples were also classified using the French–American–British (FAB) classification.^35^Table S1 reports the list of patients and detailed diagnostic data. In summary, six AML subtypes (i.e., AML M0, M1, M2, M3, M5a, and M6) and three ALL subtypes (ALL B Ph–, ALL B Ph+, and ALL T) were investigated using samples from 19 patients, each diagnosed with a distinct subtype. Representative stained cells reported in this study were stained using MayGrünwald-Giemsa (MGG) stain (Hemacolor-Merck, Germany). Part of the patients included in this study (see Table S1) were already investigated with other strategies in a previous study.^20^

### Sample Preparation

The sample preparation protocols used for this study are those used in a previous study^20^ and detailed in the Supporting Information. In brief, bone marrow aspirates from all patients were processed to isolate mononucleate cells by centrifugation and kept frozen until use. Before use, cells were washed, checked for vitality, transferred onto polylysinated CaF_2_ optical substrates, and fixed in 2% (vol) paraformaldehyde.

### Raman Measurements

All Raman measurements were performed using the home-built confocal Raman microscope used in our previous studies,^20,36^ and the details are reported in the Supporting Information. In brief, the Raman setup uses a Kr ion laser at the wavelength of 647.1 nm coupled with a modified microscope (BX41, Olympus, Japan) equipped with a dipping water objective (63*x*/1.0 NA). The Raman photons are confocally detected by a home-built spectrograph equipped with a EMCCD (1600 × 200 pixels). For cell measurements, Raman maps containing 64 × 64 (4096) spectra were collected by scanning the entire cell area, and each point spectrum was measured using 100 ms acquisition time. The optical lateral resolution was around 355 nm FWHM, and the axial resolution was around 1270 nm. The laser power measured on the sample stage was set at 35 mW.

### Preprocessing

Single spectra were first independently corrected using custom software (LabVIEW, National Instruments Corp., TX) as follows: a) cosmic ray removal by singular value decomposition (SVD);^37^ b) camera offset subtraction; c) CCD response correction (intensity and etaloning) using a tungsten halogen light with known emission (Avalight-HAL, Avantes BV, NL)); d) wavenumber calibration using the zero-wavenumber laser line, toluene and argon–mercury emission (CAL-2000, Ocean Optics, Germany); e) denoising by SVD.^37^ Before global cluster analysis a fully automated preprocessing pipeline was written in Python (*NumPy*, *SciPy*, *pandas, scikit-learn, numba, Matplotlib,* and *seaborn* modules) and applied to the entire hyperspectral data set of the 319 cells using identical parameters and including the following nine steps (see Figure S1): a) preliminary spectral truncation (550 and 3100 cm^–1^); b) map truncation (62 × 64 (3968) pixels) to remove the first two scanlines affected by high autofluorescence; c) spatial median filter (window size 3 × 3 pixels) to reduce the signal heterogeneity between neighboring pixels; d) background identification by a 4-level *k*-means clustering step (performed in the 2800–3030 cm^–1^ region) identifying as background the cluster with the lowest mean intensity. The spectrum of each cell pixel was then corrected by subtracting the median spectrum of the background. Pixels belonging to the background (>600 000 spectra) were then excluded from the final data set. Also, small (<64 pixels) isolated connected regions of foreground pixels were removed, and morphological erosion (with a 1-pixel disc) was performed on the foreground pixels to optimize cell image quality; e) Whittaker-Eilers smoother^38^ at the pixel level (second-order penalties, λ = 100); f) simultaneously with the previous step, each spectrum was put on an equidistant wavenumber grid (step size of 2 cm^–1^) over the entire spectral region; g) final spectral truncation (600–1800 and 2800–3030 cm^–1^); h) baseline correction using the *rubberband* method; (i) unit vector normalization (L^2^ norm). Before the global clustering analysis, four cells were excluded from the subsequent analysis (Table S1).

### Global Cluster Analysis and Virtual Staining

**Figure 1:**
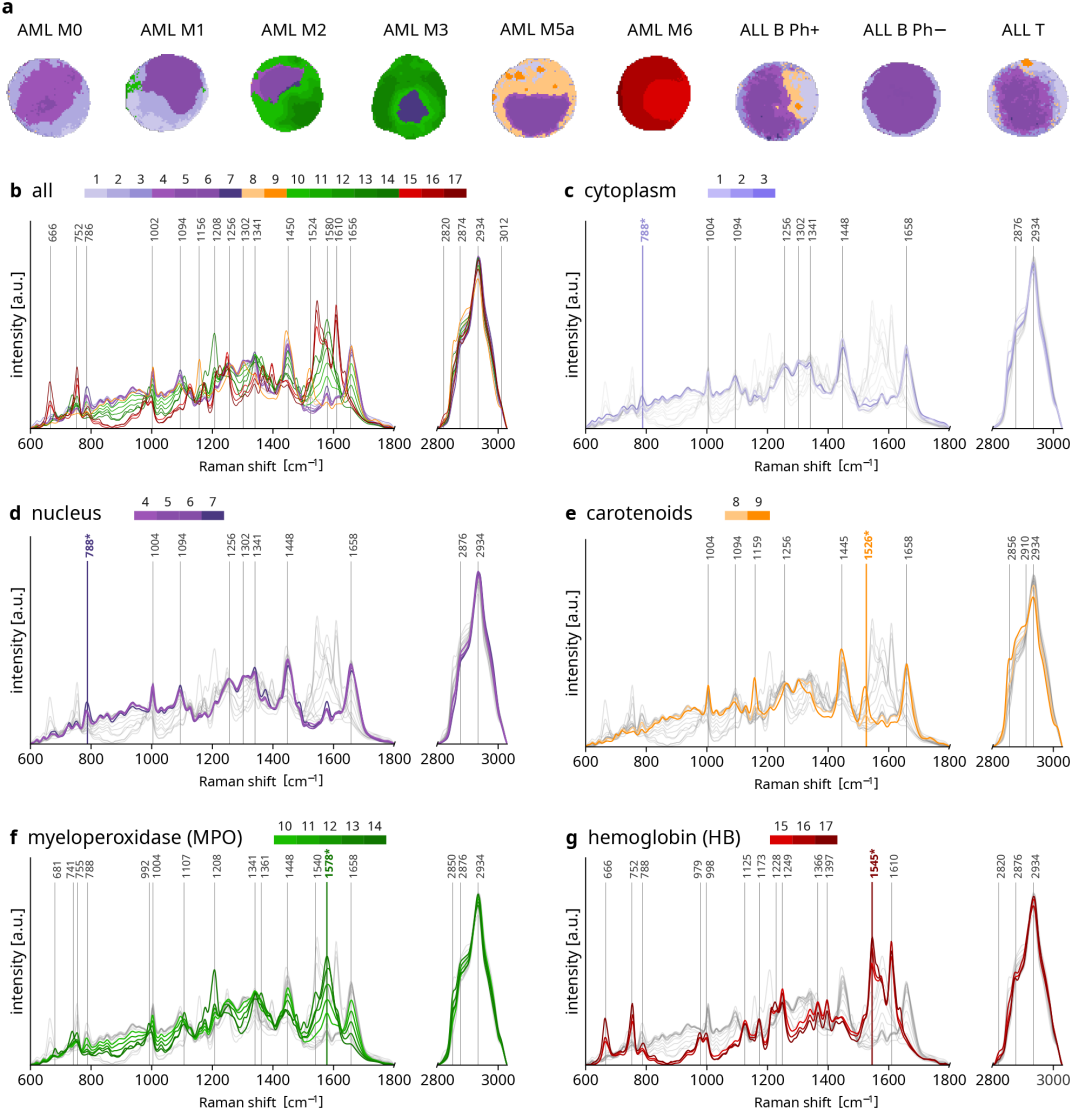
Representative Raman images of leukemia cells and their associated clusters. (a) Selected representative pseudostained Raman images of the 9 different leukemia subtypes. The colors used for Raman images are associated with different clusters as shown in panels (b)–(g), representing the centroids spectra of each of the 17 clusters and used to virtually stain the Raman images. Panel (b) includes all the 17 centroids; panels (c)–(g) include centroids associated with specific cellular components, superimposed on the remaining centroids reported in light gray for comparison. (b–g) Report Raman shifts of the most intense peaks or those better identifying each cellular component. Raman shifts with the asterisks (*) are those used to automatically select the color intensity of specific clusters.

The *k*-means algorithm was then simultaneously applied to the entire data set composed of 315 cells and 667 771 preprocessed spectra. A weighted *k*-means was employed, giving each leukemia subtype the same importance. After screening different clustering levels, the optimal number of clusters (17) was chosen to correctly identify and distinguish the main biochemical components found in leukemia cells (see the Results and Discussion section). The clusters were grouped according to the presence of Raman features associated with specific cellular components: cytoplasm, nucleus, carotenoids, myeloperoxidase (MPO), and hemoglobin (HB) (Figure S2). False-color images were produced by assigning a color to each cellular component and a different color saturation level to clusters within the same cellular component group according to the peak intensities at group-specific Raman shifts (asterisks in Figure 1).

### Classification Based on Multivariate Analysis of Global Clustering Results

The relative distribution of pixels in each cell belonging to each of the 17 clusters was computed and used as an input for linear discriminant analysis (LDA), which was used to aid in the visualization of the global clustering results at the cell level and to classify the cells according to their respective leukemia subtypes. Three main subsets were considered, namely “AMLs+ALLs”, comprising the full data set of 315 cells belonging to the nine leukemia subtypes; “AMLs” for the six AML subtypes (165 cells); “ALLs” for the three ALL subtypes (150 cells). LDA was performed on each subset separately and then followed by leave-one-cell-out cross-validation (more details in the Supporting Information).

## Results and Discussion

### Global Clustering Analysis and Virtual Staining of Leukemia Cells

Patients diagnosed with one of the nine distinct leukemia subtypes, six AMLs (i.e., AML M0, M1, M2, M3, M5a, and M6) and three ALLs (ALL B Ph–, ALL B Ph+, and ALL T) (see Table S1), were included in this study. Overall, 319 leukemia cells from bone marrow were mapped with high resolution using a home-built Raman confocal microscope using a pixel step size down to 156 nm and pixel dwell time of 100 ms. Aiming to provide final high-quality cell images reproducing those used by clinicians after specific clinical staining (i.e., May-Grünwald and Giemsa or hematoxylin and eosin protocols), we optimized a global *k*-means-based clustering analysis on the entire preprocessed final data set (667 771 spectra), thus being able to recognize the same cellular components distributed over different leukemia subtypes. Considering that clustering approaches may easily recognize spectral features and spectral intensities not directly associated with biological/pathological portraits, due to technical variability, an accurate 9-step processing pipeline (Figure S1) has been automatically and simultaneously applied before global *k*-means-based clustering analysis.

**Figure 2:**
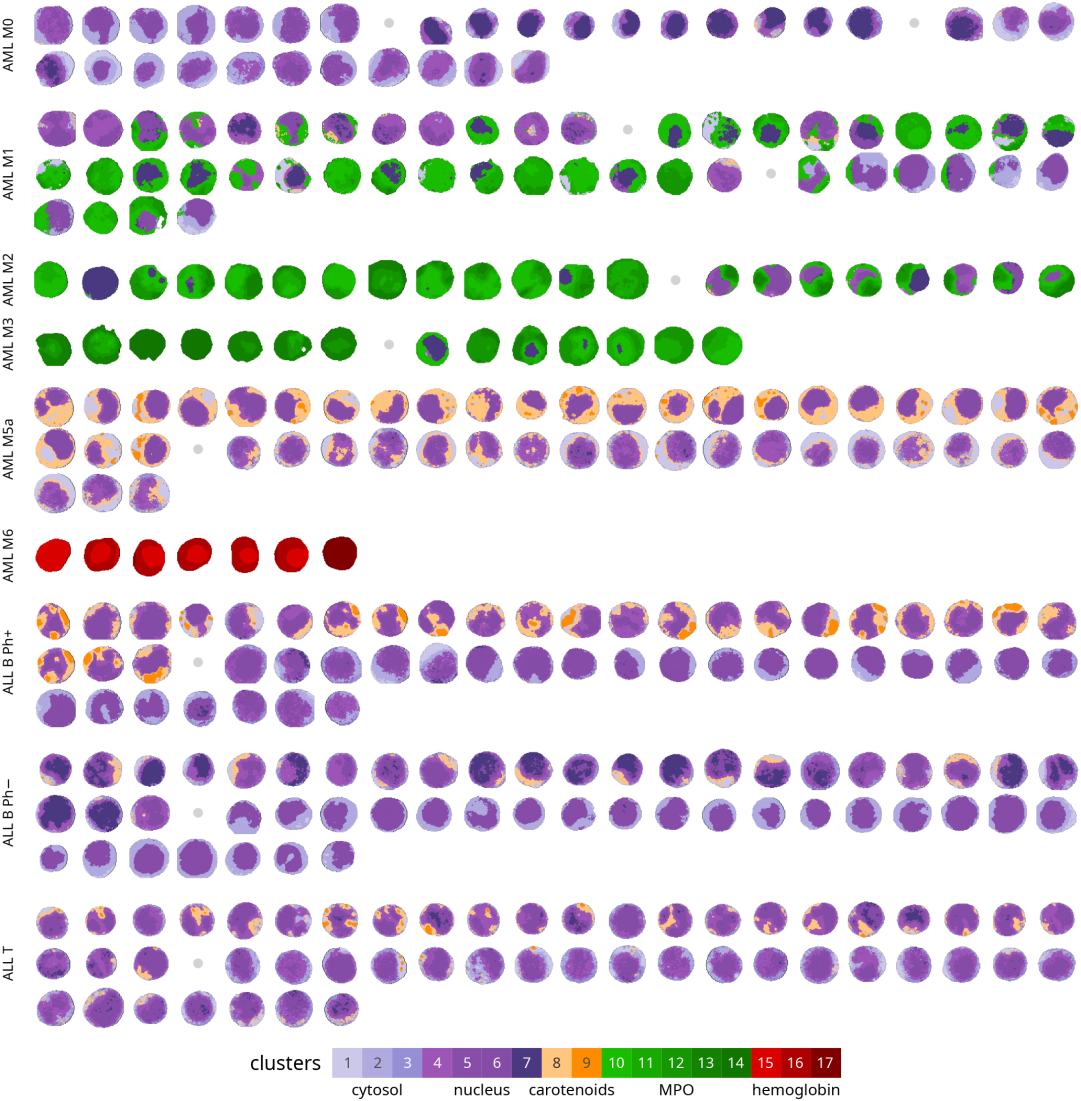
Pseudostained Raman images of 315 leukemia cells from 9 different leukemia subtypes. All Raman images were automatically preprocessed and produced by a global whole-data set cluster analysis. The colors used for Raman images are associated with different clusters as described by the legend and in Figure 1. For each subtype, cells originating from different patients are eventually separated by a gray dot, following the left-to-right, top-to-bottom direction.

Figure 1 shows representative pseudostained Raman images of cells from nine different leukemia subtypes, accompanied by the spectra representing each cluster (centroids). Figure 2 shows the entire data set including 315 cells (four cells were removed due to artifacts), pseudocolored using the same approach. The images reported in the figure were obtained by performing a 17-level global *k*-means-based clustering. The selection of the best clustering level has been performed by considering two main decisive criteria. The first criterion was to correctly detect the spectra of cellular components known to be present in leukemia cells: cytoplasm, nucleus, carotenoids, MPO, and HB. This straightforwardly follows from previously published data^20^ and from the supervised identification of these components at single pixel level (see Figure S2). The second criterion was to represent the spatial distribution and degree of concentration of specific cellular features in the cells, thus resembling the morphological features visible in clinically stained samples (Figure S3). For example, the use of 5- or 10-level clustering analysis (Figures S4 and S5) resulted in the incorrect assignment of the carotenoid cluster(s) to cells (e.g., AML M0) in which carotenoid features are rarely detected by manual spectral analysis at the single pixel level, and this was confirmed when the clustering level was increased. In addition, images resulting from a 5- or 10-level clustering analysis showed reduced intracellular complexity, missed separation between cellular compartments, or missed relative abundance of lineage-specific components.

The pseudostaining of Raman maps was performed by a semiautomatic approach. The clusters were grouped according to Raman features (peak at group-specific Raman shifts, see asterisks in Figure 1) mainly associated with five specific cellular components (cytoplasm, nucleus, carotenoids, MPO, HB) (Figure S2). Different color saturation levels within the same cellular component were assigned according to the peak intensities at the aforementioned group-specific Raman shifts. We assigned lilac (light pink) to the cytoplasm and violet to the nucleus, aiming to resemble the appearance of stained samples used in clinics (Figure S3). We then considered that staining procedures are limited in the detection of other specific molecules. For instance, MPO, an important hallmark to classify AML subtypes,^1,2^ is normally detected utilizing enzymatic-based^39^ or antibody-based^40^ labeling, on dedicated smears. Similarly, HB appears pink after staining, but also other acidic proteins may have a similar appearance, thus preventing unequivocal detection. Carotenoids can be detected only using complex (e.g., isotope-based) labeling methods^41^ or by destructive (e.g., HPLC-based) approaches,^42^ and they are not available for routine diagnosis.^43^ For these reasons, the clusters associated with MPO, HB, and carotenoids were virtually colored in green, red, and orange, respectively, to increase contrast and legibility and to eventually allow clinicians to easily recognize disease-specific biomarkers.

A direct cell-to-cell comparison between virtual staining and real staining was not possible due to difficulties in staining and locating measured cells after Raman imaging. On the other hand, most of the general morphological features (e.g., cellular shape, nuclear shape, and nuclear-cytoplasmic ratio) emerging from virtually stained cells resemble those of stained cells (Figure S3) and are aligned with morphological features reported in the available diagnostic guidelines.^1,2^

**Figure 3:**
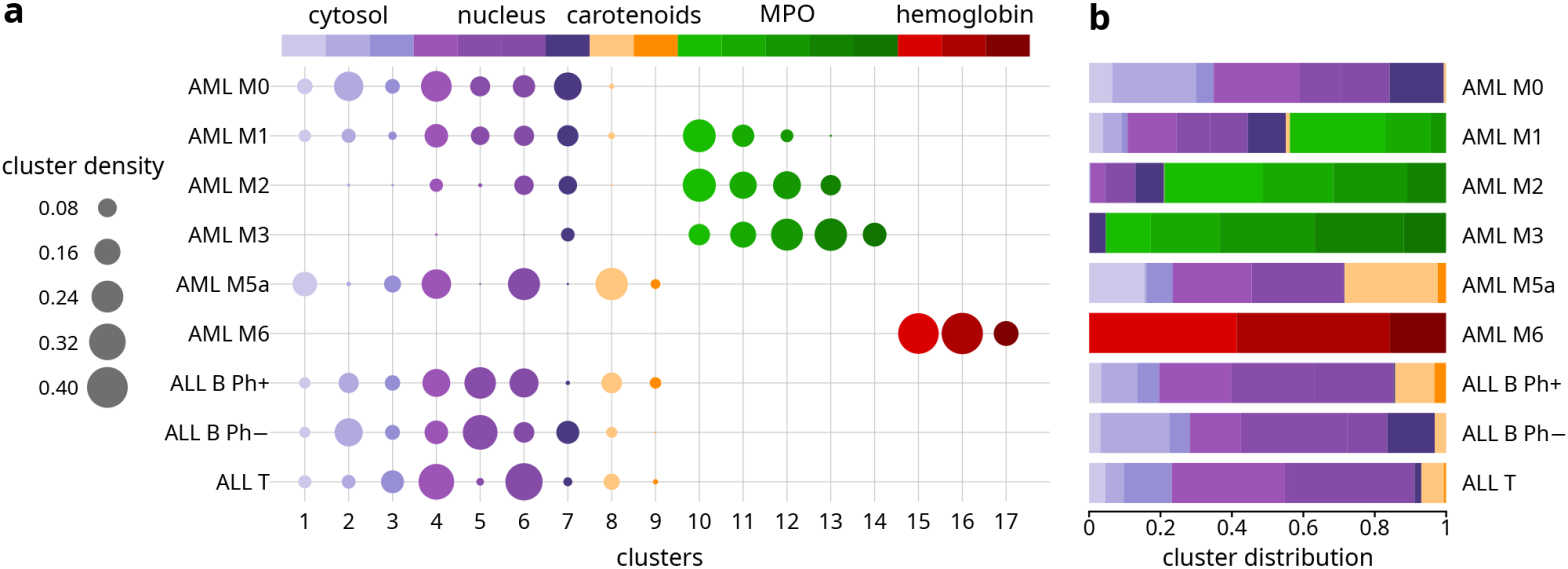
Distribution of clusters for each leukemia subtype. (a) Bubble chart showing the proportion of cellular pixels (bubble size) associated with each cluster. The legend at the top indicates the color and corresponding subcellular component of each cluster (see Figures 1 and 2). (b) Bar plot showing the same distributions, highlighting the relative cluster frequency for each leukemia subtype (see the Supporting Information for more details).

Before describing and discussing in detail the morphological and spectra information emerging from label-free pseudostained Raman images of leukemia cells, in Figure 3, vide infra, we introduce a semiquantitative analysis of the distribution of clusters—and therefore cellular components—across different leukemia subtypes, enabling a more objective description of the results presented in Figures 1 and 2.

### Raman Imaging-Based Characterization of Different Leukemia Subtypes

A general overview of Raman images (Figures 1 and 2) and cluster distribution (Figure 3) enables the description of peculiarities and common features of the two main leukemia types, namely, AMLs and ALLs. The group of five AML subtypes studied here (M0, M1, M2, M3, M5a and M6) is characterized by high complexity and high heterogeneity at both morphological and compositional levels, especially if compared to the group of the ALL subtypes (B Ph+, B Ph–, T). This is consistent with the description of leukemia subtypes by current guidelines^1,2^ and with the lineage associated with the two groups. In fact, AML affects the myeloid lineage, mainly responsible for the production of granulocytes, monocytes, and erythrocytes, which are associated with very different cellular functions, such as phagocytosis, bacteria/fungi neutralization, or oxygen transport,^44^ all associated with characteristic biochemical features (e.g., metalloproteins) and with recognizable Raman features.^20^

On the contrary, Raman images belonging to the ALL group show very similar features, generally conserved across the different ALL subtypes. This is also consistent with the biology of the lymphoid lineage, which is responsible for producing only lymphocytes, the cells that mediate humoral immunity, pathogen recognition, and the production of immune mediators (cytokines and antibodies).^45^ These cellular functions are finely regulated and intrinsically complex at the genetic and molecular levels but, compared for example to phagocytosis and oxygen transport typical of the myeloid cells, are not associated with subtle biochemical changes and therefore less visible in the Raman spectrum. In addition, as expected from official classification guidelines,^1,2^ cells isolated from ALL subtypes are very similar and can be hardly distinguished by morphological features.

When considering Raman images (Figures 1 and 2) and cluster distribution (Figure 3) of each single leukemia subtype, we can observe that AML M0, associated with an increase of undifferentiated cells (called blasts), exhibits a high nuclear-cytoplasmic ratio (see also Figure S6) and with scarce evidence of other components in the cytoplasm, which appear agranular. AML M1 Raman images are like AML M0 but show a lower nuclear-cytoplasmic ratio (Figures 1–3 and S6) and the presence of MPO signals (around 741, 992, 1028, 1540, and 1578 cm^–1^)^20,46,47^ in the cytoplasm of several cells, sometimes also prevailing over nuclear (DNA) signals (e.g., around 788 cm^–1^).^14,20,36^ MPO is a peroxidase enzyme, a metalloprotein, expressed by mature granulocytes to produce hypochlorous acid used to deactivate bacteria and other pathogens.^48^ This is consistent with the cytological description of AML M1, characterized by an increase in the myeloblast with early signs of differentiation in the samples and by MPO positivity after immunostaining.

AML M2 cells studied by Raman imaging show a further decrease in the nuclear-cytoplasmic ratio (Figure S6) and the presence of strong MPO Raman features occupying the entire cytoplasm, most of the time overlapping with the nuclear region (Figures 1–3). These signs are also consistent with diagnostic evidence related to AML M2, reporting the increase of mature cells, now called promyelocytes, and reporting MPO granules over the entire perinuclear region.

AML M3 Raman cells show very intense and largely diffuse MPO signals with morphological features like those observed in M2 but presenting a different cluster distribution. It is important to note that in M1, M2, and M3, when MPO (green) clusters colocalize also with the nuclear regions in the center of the cell, DNA signals are still visible at the spectral level (788 cm^–1^). This agrees with the presence of the nucleus in such type of cells, but since MPO granules are very abundant and their Raman signals are very strong due to preresonance effects^20,47^ the clustering analysis assigns most of the pixels to MPO-containing clusters. Moreover, as can be seen in Figure 3, MPO clusters have a different distribution over M1, M2, and M3, with the exclusive presence of cluster #14 in M3. In detail, cluster #14 (Figure 1f, dark green) shares some features with clusters #10–13, including a very strong peak at around 1578 cm^–1^, associated with MPO. At the same time, cluster #14 is characterized by a relatively high intensity of Raman peaks at around 681, 741, 992, and 1028 cm^–1^, if compared with clusters #10–13. Further studies and an enlarged population sampling will be needed to investigate the peculiarity of these spectral features in M3 cells. Guidelines associate M3 with the presence of abnormal and hypergranulated promyelocytes, described as irregular and often kidney-shaped cells, with strong MPO-staining reaction and with MPO granules that may totally obscure the nuclear cytoplasmic margins,^2^ in agreement with our data. In summary, the increasing maturation of cells, mostly represented by an increasing amount of MPO signatures and a decreasing nuclear-cytoplasmic ratio, is clearly visible when passing from AML M0 to AML M3.

When observing Raman images of AML M5a cells, the most evident feature is the presence of a variable extent of carotenoids signals in the cytoplasm, scarcely observed in the AML M0, M1, M2, and M3, and the almost complete absence of MPO. Carotenoid signals (mainly associated with bands around 1156 and 1526 cm^–1^)^49^ (Figure 1e) may occupy the entire cytoplasmic regions or be more dispersed in specific lipid-rich granules (called “gall bodies”),^50^ with a certain variability among patients. These cells are generally characterized by the abundant cytoplasm (i.e., low nuclear-cytoplasmic ratio, Figure S6) and, in some cases, it is possible to recognize typical nuclear features, including nucleoli and bilobated nuclei (Figure 2). While the nuclear and cytoplasmic features, together with the absence of MPO, are well-known features recognized by hematologists in the so-called M5a monoblasts, the presence of carotenoids is not reported as a typical sign of AML M5a in the current guidelines. This could be due to the lack of methods for the detection of carotenoids by staining or labeling procedures. At the same time, this indication points to an interesting feature that seems to characterize the M5a subtype, at least among the AML subtypes. As of now, no previous literature has reported a correlation between subcellular carotenoid deposits and AML M5a leukemia. This suggests that label-free imaging can not only guide leukemia classification but also offer new fundamental insights into its subtypes.

AML M6 cells studied by Raman imaging approaches reveal very strong HB signals, diffused over the entire cellular volume, and characterized by very sharp and very specific preresonance Raman features (mainly around 665, 752, 1545, and 1610 cm^–1^)^20,51^ (Figure 1g), all associated with the heme group (porphyrin). The presence of HB agrees with classification guidelines reporting the predominance of erythroid precursors (erythroblasts) in patients’ samples.^1−3^ At the same time, even if the preponderance of hemoglobin prevents nuclear pixels from being automatically assigned to nuclear clusters (#4–7), the shape of the nucleus can be recognized in some AML M6 cells, described by cluster #15, and also DNA signals (788 cm^–1^) are visible at the spectral level (Figure 1). For instance, the nuclear presence is in agreement with the description of erythroid precursors in AML M6.^1−3^

Concerning ALL subtypes, the cluster analysis produced similar Raman images for all three subtypes (ALL B Ph+, ALL B Ph–, and ALL T) with a certain degree of variability among cells and, even more pronounced, among patients. In general, most of ALL cells (lymphoblasts) revealed a high nuclear-cytoplasmic ratio (Figure S6)—like what was observed in AML M0 cells—and the complete absence of MPO. This agrees with the definition of ALL subtypes, belonging to the lymphoid lineage and lacking MPO production.^1−3^ At the same time, some cells, especially those belonging to specific patients, showed carotenoid signals, like what was observed in AML M5a cells, but generally less abundant. As observed for AML M5a cells, also in the case of ALL subtypes, the presence of carotenoids and their correlation with leukemia are not reported in the current guidelines. What is clear from our data is that carotenoids cannot be distinctive of a single leukemia subtype. At most, our data suggest that carotenoids can be a possible biomarker of AML M5a when considering only the AML group. When comparing ALL B vs ALL T, or ALL B Ph+ vs ALL B Ph– vs ALL T, there are no clear differences from the morphological and qualitative evaluation of global clustering results.

### Multivariate Analysis of Global-Clustering Results

**Figure 4:**
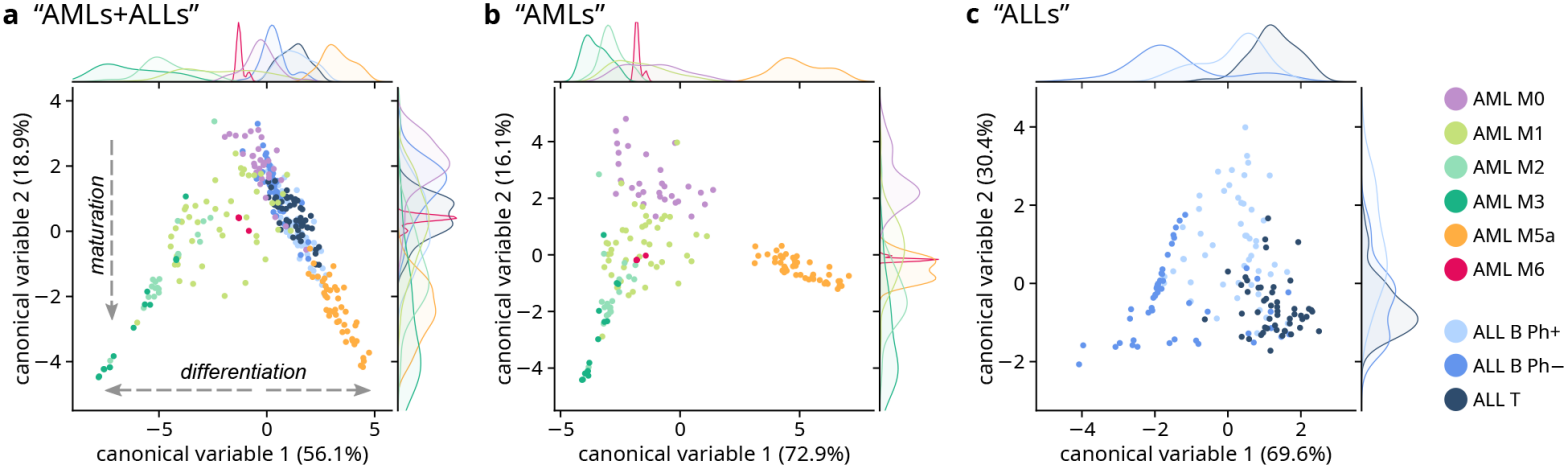
Multivariate analysis of the global-clustering analysis results, using the cluster distributions for each cell as an input. Scatter plot of the first two scores of LDA of the cluster distribution in cells from (a) all nine leukemia subtypes (“AMLs+ALLs”); (b) AML leukemia subtypes (“AMLs”); c) ALL leukemia subtypes (“ALLs”). Kernel density estimate (KDE) plots are reported at the top/right of each scatter plot to better show the marginal distributions of, respectively, CV1 and CV2. Percentage values in parentheses represent the proportions of variance explained by the corresponding CV. The scatter plots of all the CVs are reported in Figures S7 and S8 for “AMLs+ALLs” and “AMLs”; the scalings for the three LDAs are reported in Figure S9. The KDEs relative to AML M6 in panels (a) and (b) have been rescaled for visualization purposes.

Linear discriminant analysis (LDA) was applied to cluster distribution results, used as unique input, rather than using spectral features as traditionally done in the literature. This starts from the hypothesis that the distribution of subcellular features—represented by 17 clusters—can be associated with different leukemia subtypes. The resulting scatter plots, reporting the scores of the first two canonical variables (CVs) for each LDA, are shown in Figure 4a–c. The scatter plots including all of the calculated CVs are reported in Figures S7 and S8 for “AMLs+ALLs” and “AMLs”, respectively (LDA of “ALLs” produced only 2 CVs, with data already reported in Figure 4c). The scalings (also called LDA coefficients or loadings), providing the interpretation about the importance of different clusters in the separation of different cells and subtypes, were calculated for the three LDAs and reported in Figure S9.

As shown in Figures 4a and S7, representing scores of all nine leukemia subtypes after LDA of “AMLs+ALLs”, a certain degree of separation was obtained between different subtypes. Clear maturation and differentiation trends can also be observed (see arrows in Figure 4a). More in details, the decrease of CV2 scores (to the bottom side of the scatter plot) describes the maturation trend of leukemia cells: from M0 (undifferentiated leukemia cells, called “blasts”) to more mature cells in both the myeloid (i.e., AML M1, M2, M3, M5a and M6) and the lymphoid (i.e., ALL B and T) lineages. Instead, CV1 scores describe the differentiation trend of leukemia cells toward clearly different phenotypes: negative CV1 scores bring to promyelocytes (from AML M1 to M3); positive CV1 scores bring to lymphoblasts (ALLs) and monoblasts (AML M5a). Despite being a supervised method, the fact that LDA provided valuable insights into the ordering of leukemia subtypes by maturation and differentiation status—even though this information was not explicitly provided to the algorithm—demonstrates the validity and robustness of the method, suggesting that LDA of clustering results captured underlying patterns beyond the explicitly labeled class information. The differentiation toward erythroblasts (AML M6) is only marginally described by CV2 scores (close to zero) but this is clearly visible when considering scores of CV3 and CV4 (Figure S7). In parallel, scalings related to CV1 (Figure S9a) show the importance of MPO-related clusters (negative CV1 values) and of carotenoid-related clusters (positive CV1 values) in the description of leukemia cell differentiation toward promyelocytes or toward monoblasts, respectively. As confirmation, the confusion matrix produced by the LDA of clustering results (Table S2, “AMLs+ALLs”) shows good classification performances associated with AML M5a and M6 (*F*_1_-scores of 0.87 and 1, respectively). A certain degree of separation can be seen between M0, M1, M2, and M3 but these are sometimes misclassified (*F*_1_-scores between 0.64 and 0.68; Table S2), and this is mainly due to the presence of MPO clusters in these four diseases. ALL B Ph+ and ALL B Ph– are generally misclassified (*F*_1_-scores 0.57 and 0.58, respectively) but ALL T can be partially distinguished from ALL Bs (*F*_1_-score 0.70, Table S2). ALL subtypes are subject to differential diagnoses and misinterpretation also in clinical practice,^2^ as distinguishing between ALLs by visual and morphological criteria is not always straightforward.

When only AML subtypes are considered, LDA can separate AML M0, M5a, M6, and the cloud of M2/M3 cells (“AMLs”; Figures 4b and S8). Again, the maturation stage is associated with CV2 and the differentiation is associated with CV1 (see also scalings in Figure S9b). The confusion matrix resulting from this LDA (Table S2, “AMLs”) shows very good accuracy for the classification of AML M5a and M6 (*F*_1_-score 1 for both) and for the classification of AML M0 (*F*_1_-score 0.86). Again, in Figure 4b M2 and M3 are partially overlapped (*F*_1_-scores of 0.71 and 0.67, respectively) and M1 (*F*_1_-scores of 0.80) sits between M0 and M2/M3 (Table S2). As said before, this is aligned with the maturation stage of AML M1 cells, which is halfway between M0 (undifferentiated cells) and M2/M3 (promyelocytes at a higher maturation stage).

When only ALL subtypes are analyzed by LDA (“ALLs”; Figure 4c) a certain separation can be seen between the three subtypes, especially between ALL B Ph– and ALL T (*F*_1_-scores of 0.76 and 0.73, respectively). CV1, representing most of the between-class variation in ALLs, is characterized by scalings (Figure S9c) reporting variance mainly between different nucleus-related clusters. In detail, it seems that the DNA-related cluster #7 (negative CV1 values) is specifically associated with ALL B Ph–. This is also visible in false-color images of ALL cells (Figure 2) and in the centroids reported in Figure 1, showing a higher intensity of DNA-related peaks at 788 cm^–1^. The variance associated with CV2 seems not to be relevant for the separation between ALL subtypes and, according to scalings (Figure S9c), this variance is represented by the presence of carotenoids whose distribution among cells and patients seems to be not associated with a specific ALL subtype.

Finally, two other LDAs have been performed aiming to assess the possibility to discriminate different leukemia macro categories. When AML M0 cells (unmature leukemia cells), AML M1+M2+M3 grouped together (maturing promyelocytes) and AML M5a cells (maturing monoblasts), were analyzed by LDA, *F*_1_-scores of 0.88, 0.95, and 1 were obtained (Table S2), with an overall accuracy of 0.95. This is aligned with what was seen above and suggests clear biochemical differences among these three macro categories. When all AMLs and all ALLs cells were gathered in two macro groups and analyzed by LDA, the confusion matrix provided an *F*_1_-score of 0.81 (Table S2), for both classes, showing the potential of separating ALLs and AMLs, even if these two classes are intrinsically heterogeneous and constituted by different subtypes.

## Conclusions

In this study, starting from Raman imaging data, an optimized single-step global clustering approach applied to a data set of 315 cells from patients affected by nine different leukemia subtypes facilitated the automatic generation of high-quality false-color images and offered a direct depiction of the intricate biochemical and morphological features of cells. Using a semiautomatic approach, we virtually stained each cluster to replicate images commonly examined by pathologists. Beyond highlighting the cytoplasm (pink) and nucleus (violet) contrast provided by MGG staining methods, we introduced specific color representations for carotenoids, MPO, and HB, typically undetected by standard staining procedures, potentially serving as valuable subtype-specific hallmarks.

The global clustering approach provided high-quality imaging, often displaying submicrometric spatial features consistent with those described in classification guidelines (e.g., variation of nuclear-cytoplasmic ratios among different subtypes, localization of specific biomarkers (MPO)) or suggesting new possible subtype-specific features (e.g., carotenoids)). Moreover, this approach enabled a semiquantitative and objective characterization of a substantial amount of hyperspectral data. Visual interpretation (images and bar plots) and multivariate analysis (LDA) unveiled maturation and differentiation trends across various leukemia subtypes, particularly highlighted by MPO, carotenoids, and HB expression. Encouraging results were observed in classification, especially for AML M0, AML M6, and AML M5a, as well as when grouping major leukemia categories. To the best of our knowledge, this study represents the most extended Raman characterization of leukemia subtypes from patients but also the most extended spontaneous Raman imaging study of single cells from patients, reporting images from >300 cells, analyzed using a single clustering analysis step.

Crucially, meticulous data preprocessing, minimizing experimental variability, played a pivotal role in this process. To address this issue, we implemented an automatic preprocessing pipeline applied to the entire data set, eliminating the need for manual or sample-specific interventions. Interestingly, the definition and optimization of the above-mentioned preprocessing pipeline triggered the development of a web-based free tool (“RamApp”) for the preprocessing and intuitive analysis of hyperspectral data.^52^

It is also important to say that, even considering the large amount of data analyzed here, we included a limited number of patients and cells for each subtype, as well as a relatively high number of different subtypes, preventing the validation of a Raman-based classification of leukemia subtypes using training and test data sets or “leave-one-patient-out” strategies. A larger number of cells and patients per subtype would have enhanced the validation and allowed for the development of a more accurate classification model, but availability of patient’s samples and time-demanding procedures for data acquisition played an important role. For instance, the collection of high-resolution Raman maps is currently hindered by the intrinsic slow acquisition speed of standard spontaneous Raman approaches, limiting the amount of data that can be gathered. New approaches, such as coherent Raman microscopy,^9^ eventually coupled with microfluidic approaches,^53,54^ hold promise in improving the speed and potential of Raman techniques.

In conclusion, our study demonstrates the ability of hyperspectral Raman data to yield highly informative false-color images and classification, when processed by a single-step pixel-level global clustering analysis over the entire data set. The potential of this approach extends beyond leukemia, providing a versatile tool to unravel complex spatial and molecular features in diverse cellular investigations and opening new avenues for impactful biomedical research.

## Data Availability

The data underlying this study are openly available in Zenodo repository at https://zenodo.org/doi/10.5281/zenodo.11195903.

## Supporting Information Available

Sample preparation, Raman measurements, analysis of cluster distribution, patient’s characteristics (Table S1), confusion matrices (Table S2), scheme (Figure S1), single pixel spectra of cellular components (Figure S2), comparison with stained cells (Figure S3), 5-level cluster analysis (Figure S4), 10-level cluster analysis (Figure S5), nuclear-to-cytoplasmic ratios (Figure S6), LDA scatter plots for “AMLs+ALLs”) (Figure S7), LDA scatter plots for “AMLs” (Figure S8), LDA scalings (Figure S9) (PDF)
