# Supplementary material for: High-Resolution Raman Imaging of >300 Patient-Derived Cells from Nine Different Leukemia Subtypes: A Global Clustering Approach
Source: Anal Chem. 2024 May 31;96(23):9468–77. doi: 10.1021/acs.analchem.4c00787 (PMC11170555; doi:10.1021/acs.analchem.4c00787)
Supplement: Supplementary file 1 — ac4c00787_si_001.pdf [file ac4c00787_si_001.pdf]

Supplementary Information

High-resolution Raman imaging of >300 patient-derived cells from nine different leukemia subtypes: a global clustering approach

Renzo Vanna[1]*‡, Andrea Masella[2]‡, Manuela Bazzarelli[2], Paola Ronchi[3], Aufried Lenferink[4], Cristina Tresoldi[3], Carlo Morasso[5], Marzia Bedoni[6], Giulio Cerullo[1,7], Dario Polli[1,7], Fabio Ciceri[3], Giulia De Poli[2], Matteo Bregonzio[2], Cees Otto[4].

1 Istituto di Fotonica e Nanotecnologie – Consiglio Nazionale delle Ricerche (IFN-CNR), c/o Politecnico di Milano, Milan 20133, Italy; 2 Datrix S.p.A., Milan 20121, Italy; 3 IRCCS Ospedale San Raffaele, University Vita-Salute San Raffaele, Milan 20132, Italy; 4 Medical Cell BioPhysics, Department of Science and Technology, TechMed Center, University of Twente, Enschede NL 7500 AE, The Netherlands; 5 Istituti Clinici Scientifici Maugeri IRCCS, Via Maugeri 4, Pavia 27100, Italy; 6 IRCCS Fondazione Don Carlo Gnocchi, Milan 20148, Italy; 7 Politecnico di Milano, Dipartimento di Fisica, Milan 20133, Italy.

* Corresponding author: renzo.vanna@cnr.it; ‡These authors contributed equally.

TABLE OF CONTENT:

Sample preparation 3

Raman measurements 3

Analysis of cluster distribution 3

Table S1 (patient's characteristics) 4

Table S2 (confusion matrices) 5

Figure S2 (single pixel spectra of cellular components): 7

Figure S3 (comparison with stained cells): 8

Figure S4 (5-level cluster analysis): 9

Figure S5 (10-level cluster analysis): 10

Figure S6 (nuclear-cytoplasmic ratios): 11

Figure S7 (LDA scatter plots for "AMLs+ALLs): 12

Figure S8 (LDA scatter plots for "AMLs"): 13

Figure S9 (LDA scalings): 14

REFERENCES 14

Sample preparation

The sample preparation protocols used for this study were optimized in a previous study [1]. Bone marrow aspirates from all patients were processed to isolate mononucleate cells by Ficoll-Hypaque (Pharmacia Biotech, Sweden) density gradient. Cells were then resuspended in fetal bovine serum (FBS) including DMSO 10 %. Around 2 mL (1×10^6 cells/mL) of suspension were then immediately frozen and stored in liquid nitrogen until their use. Before use, the cell suspension was thawed at 37 °C and diluted in 10 mL of warm RPMI1640 (10 % FBS, 1 % PenStrep, 1 % L-Glu). Cells were then washed twice in warm PBS (400 RCF for 10 min) and resuspended in the same medium (1×10^6 cells/mL). Cells vitality was confirmed by trypan blue dye exclusion as >90 %. In parallel, CaF2 optical substrates (20 mm diam.) (Crystran LTD, UK) were incubated for 30 min at 37 °C (or overnight at 4 °C) in poly-lysine 0.01 % and then washed in PBS. 1 mL of cell suspension was trans-ferred onto the polylysinated substrate and incubated for 30 min at 37 °C. Cells were rinsed three times with warm PBS and fixed in warm 2% (vol) paraformaldehyde-PBS solution for 30 min at 37 °C. After three washing steps the CaF2 disc was quickly transferred into a 35 mm culturing polystyrene petri dish, fixed onto the petri dish using a small drop of neutral nail polish, covered with a few mL of PBS and immediately measured at the Raman microscope.

Raman measurements

All Raman measurements were performed using the home-built confocal Raman microscope used in our previous studies [1,2]. The Raman setup uses a Kr ion laser (Innova 70C-Spectrum, Coherent, USA) at the wavelength of 647.1 nm as the excitation source. The laser is filtered with a band-pass filter (LL01-647-12.5, Semrock, USA) and reflected by a dichroic beam splitter (Di02-R635, Semrock, USA). The light passes a 4F lens-system with a beam expanding property of 2, used for beam scanning, before reaching a modified microscope (BX41, Olympus, Japan) that focuses the light on the sample by a dipping water objective (63x/1.0 NA, Zeiss, Germany). The photons coming back from the sample are collected by the objective and then transmitted by the dichroic beam splitter and filtered to remove the excitation light thanks to a razor-edge long-pass filter (LP02-647RU, Semrock, USA). At this point, the Raman photons are focused by a lens (f = 30 mm, AC127-030-B, Thor Labs, Newton, NJ, USA) on a confocal pinhole with a diameter of 15 µm positioned before the spectrograph, with final magnification equal to 23x. The Raman photons are detected by a home-built spectrograph equipped with a thermoelectrically cooled electron-multiplying charge-coupled device (EMCCD) chip (1600×200 pixels, image area: 25.6 mm×3.22 mm) (Newton DU-970N, Andor Technology, Northern Ireland). The resulting spectral range corresponds to around 3600 cm−1 with an average spectral dispersion of 2.25 cm^{-1}. For cell measurements, Raman maps containing 64×64 (4096) spectra were collected by scanning the entire cell area with a galvanometric scanning mirror (Leica, Germany). The physical size of the mapped area varied according to cellular size and was between 10 and 14 µm, thus obtaining a step size between 156 and 218 nm. In any case, the optical lateral resolution was around 355 nm FWHM and the axial resolution was around 1270 nm. The laser power measured on the sample stage was set to 35 mW. Each point was measured using 100 ms as spectral acquisition time, thus resulting in a total acquisition time of 6.8 min for each mapped cell, independently of the map size. For each cell, the focus was set between 3 and 7 µm above the CaF_2 disc surface, depending on the cell size.

Analysis of cluster distribution

Following the global clustering, the number of pixels belonging to each of the 17 clusters was aggregated for each cell and each subtype, providing cell- and subtype-level distributions of the clusters. The subtype-level distributions (normalized per subtype) were used to characterize the different leukemia subtypes (Fig. 3). The normalized cell-level distributions, representing the relative percentage of pixels in each cell belonging to each of the 17 clusters, were used for linear discriminant analysis (LDA) (Fig. 4).

Table S1 (patient's characteristics). *FAB subtypes names, M0: "AML with minimal evidence of myeloid differentiation"; M1: "AML without maturation"; M2: "AML with maturation"; M3: "Acute hypergranular promyelocytic leukemia"; M5a: "Acute monoblastic leukemia"; M6: "AML with predominant erythroid differentiation"; ALL B Ph+: "Philadelphia-Positive B Acute Lymphoblastic Leukemia"; ALL B Ph−: "Philadelphia-Negative B Acute Lymphoblastic Leukemia"; ALL T: "T Acute Lymphoblastic Leukemia". Abbreviations: FAB, French-American-British classification; AML, acute myeloid leukemia; APL, acute promyelocytic leukemia; NOS, not otherwise specified (without currently known recurrent genetic abnormalities). 4 cells (in parentheses) were excluded from the results shown here due to severe artefacts (2 cells) or due to the presence of spurious cells in the selected fields of view (2 cells). (*) Cells analyzed in a previous study [1].*

Patient n.	Leukemia Subtype (FAB Classification)	WHO Classification	Typical cellular subpopulation	Age at the diagnosis	Sex	Percentage of blasts	Cells (excluded from final analysis)
1	**AML M0**	AML with minimal differentiation, NOS	blasts	83	F	97	7*
2	**AML M0**	AML with minimal differentiation, NOS	blasts	70	M	85	10*
3	**AML M0**	AML with mutated c/EBPA	blasts	71	F	91	14*
4	**AML M1**	AML w/o maturation, NOS	myeloblasts	50	F	94	12
5	**AML M1**	AML w/o maturation, NOS	myeloblasts	61	F	86	25 (1)
6	**AML M1**	AML w/o maturation, NOS	myeloblasts	70	M	63	10
7	**AML M2**	AML with t(8;21) ; RUNX1-RUNX1T1	promyelocytes	65	M	32	13*
8	**AML M2**	AML with t(8;21) ; RUNX1-RUNX1T1	promyelocytes	60	M	51	8*
9	**AML M3**	APL with t(15;17); PML-RARA	abnormal promyelocytes	23	M	88	7*
10	**AML M3**	APL with t(15;17); PML-RARA	abnormal promyelocytes	36	M	n.a.	7*
11	**AML M5a**	Acute Monoblastic Leukemia, NOS	monoblasts	81	F	82	25
12	**AML M5a**	Acute Monoblastic Leukemia, NOS (therapy related)	monoblasts	65	F	89	24 (3)
13	**AML M6**	Acute Erythroid Leukemia, NOS	erythroblasts	64	M	n.a.	7*
							169 (4); 165
14	**ALL B Ph+**	ALL B with t(9;22)(q34;q11.2); BCR-ABL1	blasts	64	F	80	25
15	**ALL B Ph+**	ALL B with t(9;22)(q34;q11.2); BCR-ABL1	blasts	50	M	88	25
16	**ALL B Ph−**	B lymphoblastic leukemia/lymphoma, NOS	blasts	82	F	89	25
17	**ALL B Ph−**	B lymphoblastic leukemia/lymphoma, NOS	blasts	50	M	99	25
18	**ALL T**	T lymphoblastic leukemia/lymphoma, NOS	blasts	54	F	89	25
19	**ALL T**	T lymphoblastic leukemia/lymphoma, NOS	blasts	46	M	91	25
							150
							**319 (4); 315**

Table S2 (confusion matrices). *Confusion matrices and figures of merits of the LDA classification results (using leave-one-cell-out cross-validation) of leukemia subtypes based on the cell pixel distributions across clusters, for each of the considered subsets.* ***a****) "AMLs+ALLs";* ***b****) "AMLs";* ***c****) "ALLs";* ***d****) "AML M0 vs AML M1/M2/M3 vs AML M5a";* ***e****) "AMLs vs ALLs".*

a "AMLs+ALLs"

true \ predicted	AML M0	AML M1	AML M2	AML M3	AML M5a	AML M6	ALL B Ph+	ALL B Ph−	ALL T	precision (PPV)	recall (sensitivity)	F_1-score
AML M0	22	0	0	0	1	0	0	8	0	0.59	0.71	0.65
AML M1	6	27	3	1	0	0	2	4	3	0.79	0.59	0.68
AML M2	1	4	14	2	0	0	0	0	0	0.70	0.67	0.68
AML M3	0	3	3	8	0	0	0	0	0	0.73	0.57	0.64
AML M5a	0	0	0	0	42	0	2	0	2	0.82	0.91	0.87
AML M6	0	0	0	0	0	7	0	0	0	1.00	1.00	1.00
ALL B Ph+	1	0	0	0	7	0	25	9	8	0.66	0.50	0.57
ALL B Ph−	6	0	0	0	0	0	3	30	11	0.57	0.60	0.58
ALL T	1	0	0	0	1	0	6	2	40	0.62	0.80	0.70

b "AMLs"

true \ predicted	AML M0	AML M1	AML M2	AML M3	AML M5a	AML M6	precision (PPV)	recall (sensitivity)	F_1-score
AML M0	27	4	0	0	0	0	0.84	0.87	0.86
AML M1	4	38	3	1	0	0	0.78	0.83	0.80
AML M2	1	4	15	1	0	0	0.71	0.71	0.71
AML M3	0	3	3	8	0	0	0.80	0.57	0.67
AML M5a	0	0	0	0	46	0	1.00	1.00	1.00
AML M6	0	0	0	0	0	7	1.00	1.00	1.00

c "ALLs"

true \ predicted	ALL B Ph+	ALL B Ph−	ALL T	precision (PPV)	recall (sensitivity)	F_1-score
ALL B Ph+	29	8	13	0.67	0.58	0.62
ALL B Ph−	6	36	8	0.80	0.72	0.76
ALL T	8	1	41	0.66	0.82	0.73

d "AML M0 vs AML M1/M2/M3 vs AML M5a"

true \ predicted	AML M0	AML M1/M2/M3	AML M5a	precision (PPV)	recall (sensitivity)	F_1-score
AML M0	28	3	0	0.85	0.90	0.88
AML M1/M2/M3	5	76	0	0.96	0.94	0.95
AML M5a	0	0	46	1.00	1.00	1.00

e "AMLs vs ALLs"

true \ predicted	AML	ALL	precision (PPV)	recall (sensitivity)	F_1-score
AML	126	39	0.85	0.76	0.81
ALL	22	128	0.77	0.85	0.81

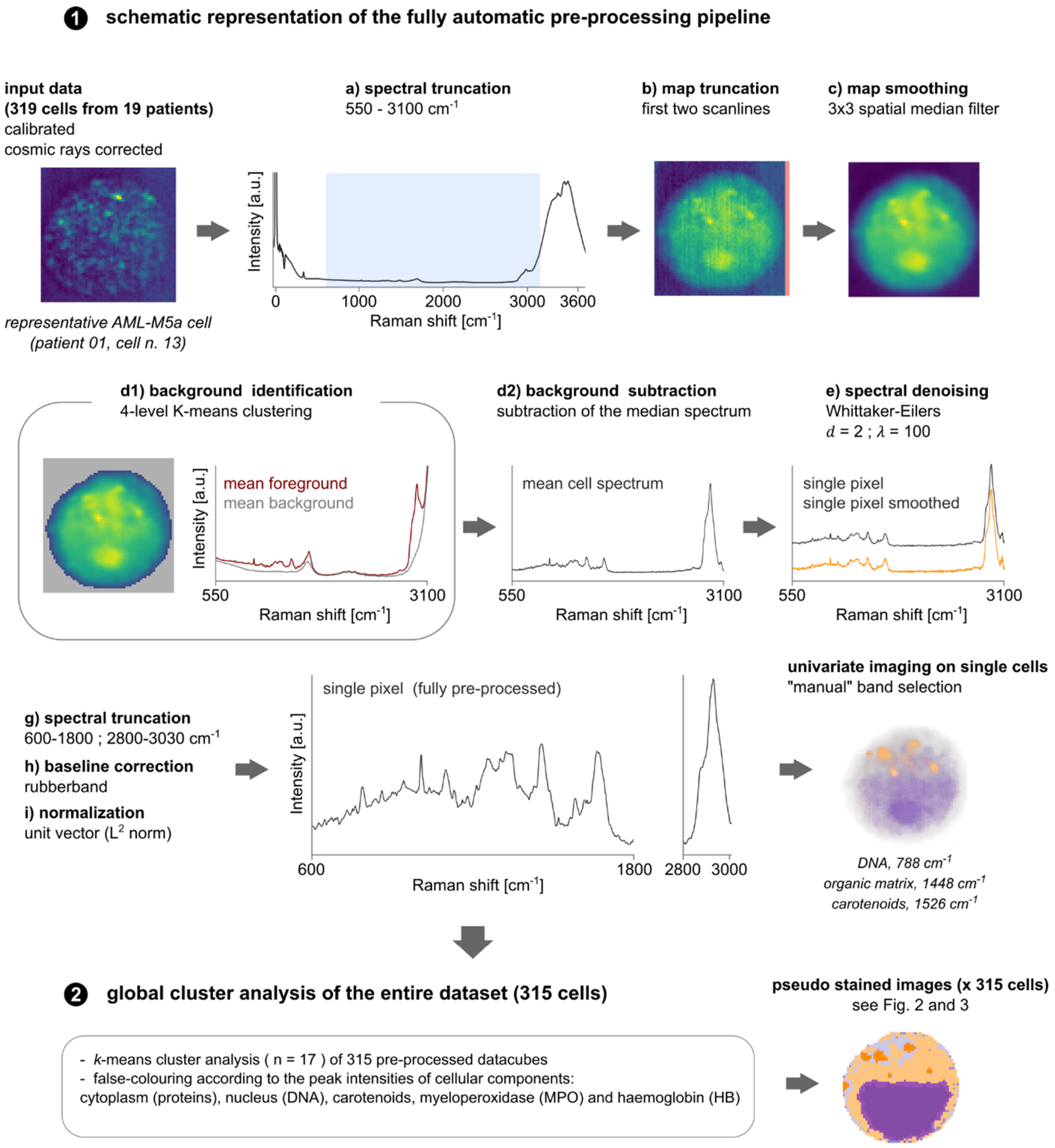

Figure S1 (scheme)*: Schematic representation of the data analysis strategy used in this study (see "Experimental section" for more details).*

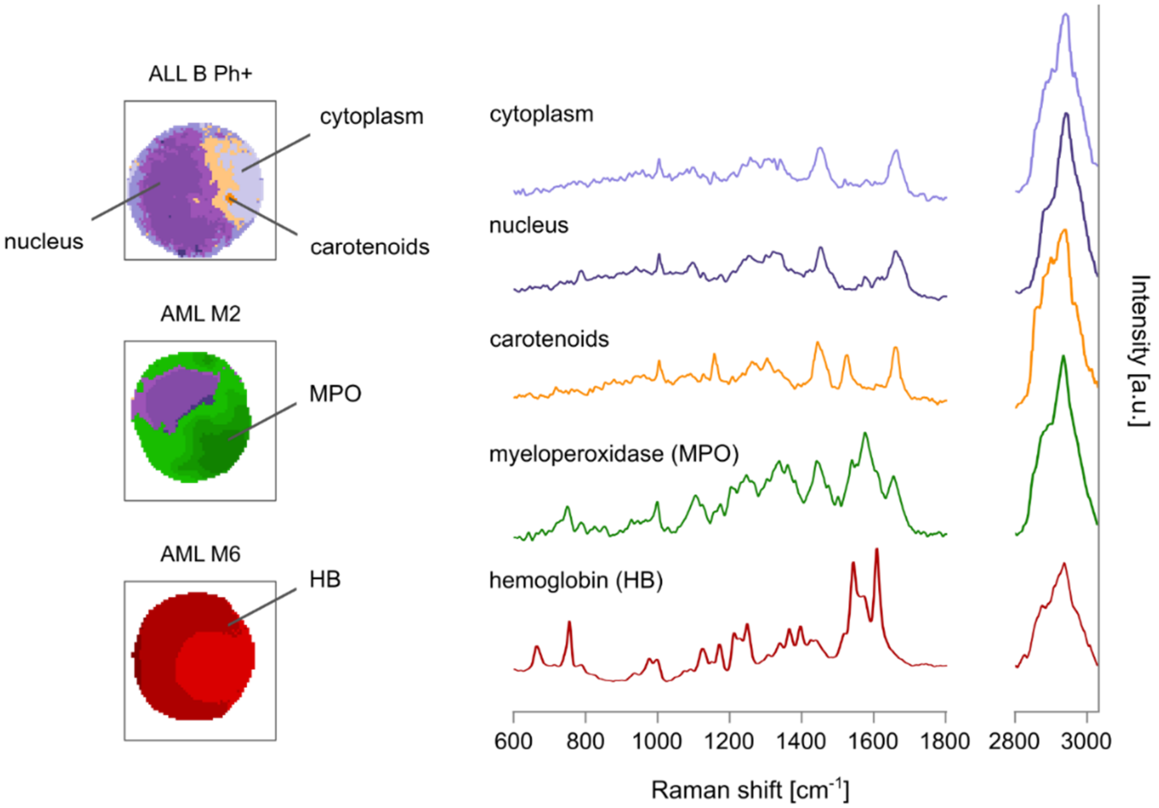

Figure S2 (single pixel spectra of cellular components): *representative examples of spectra collected at single-pixel level after the preprocessing pipeline described in the manuscript, in correspondence of different cellular components. The spectra reported on the right correspond to the pixels indicated on the false-color images on the left. Spectra were shifted for clarity.*

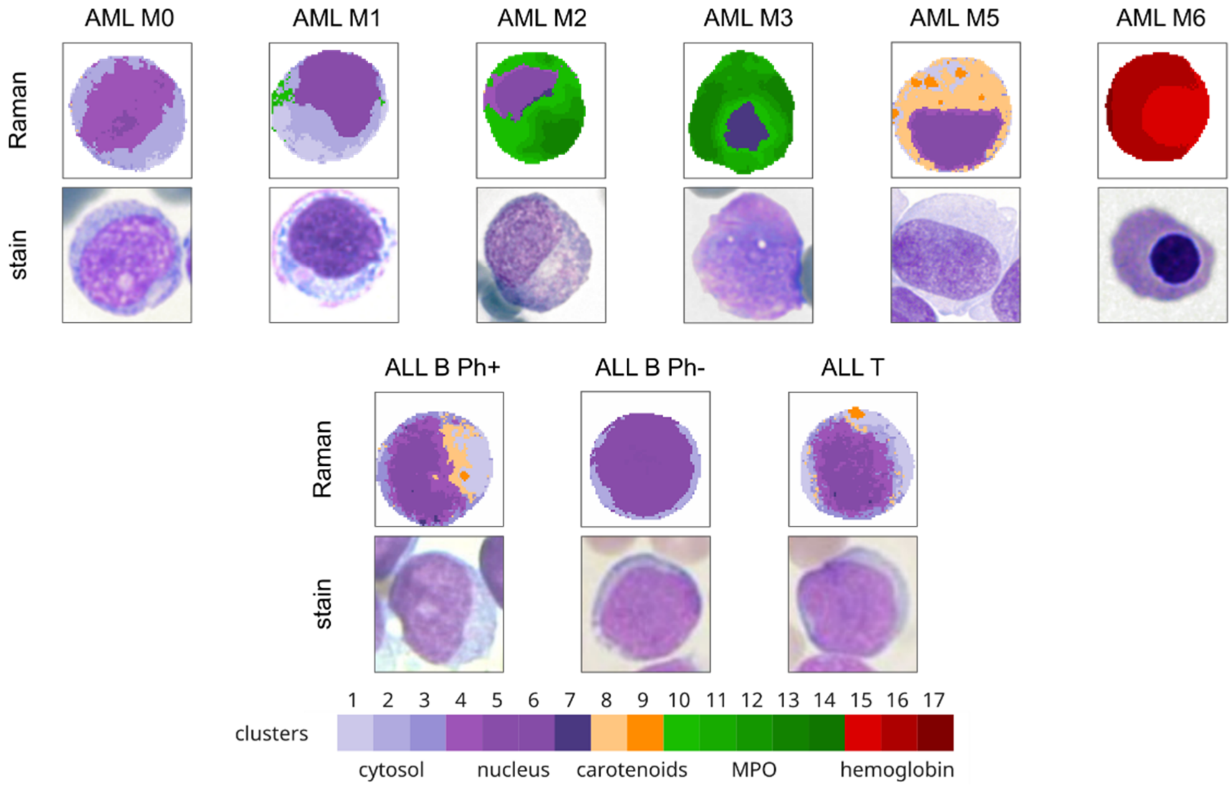

Figure S3 (comparison with stained cells): *selected representative pseudo-stained Raman images of the 9 different leukemia subtypes (top, as reported in Fig. 1) accompanied by representative images of cells stained using May-Grünwald and Giemsa protocol (bottom). Images of stained cells, used here for comparison, were not collected from the same cell analyzed by Raman imaging.*

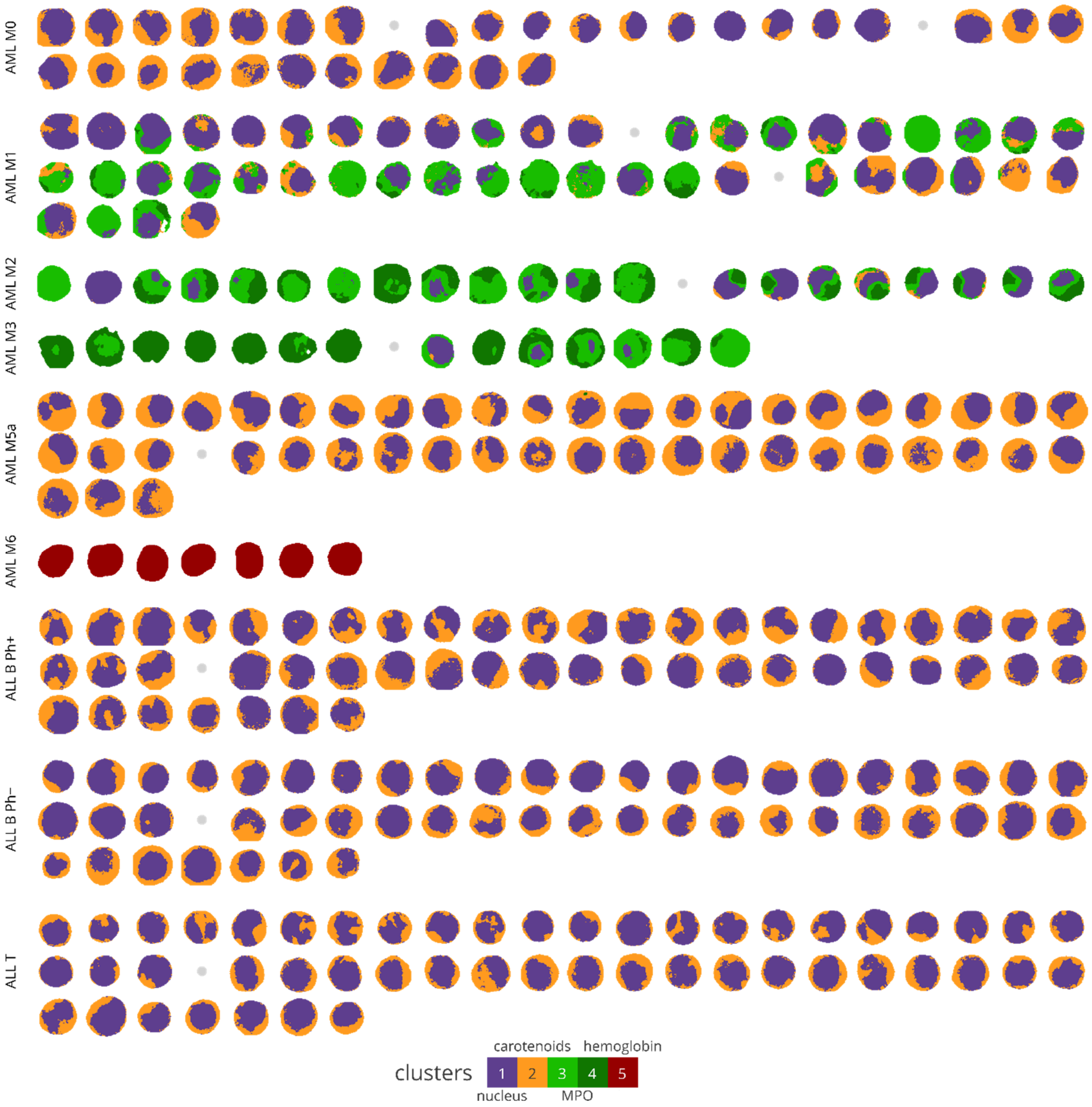

Figure S4 (5-level cluster analysis): *Pseudo-stained Raman images of 315 leukemia cells from 9 different leukemia subtypes, produced by a global whole-dataset cluster analysis limited to 5 clusters.*

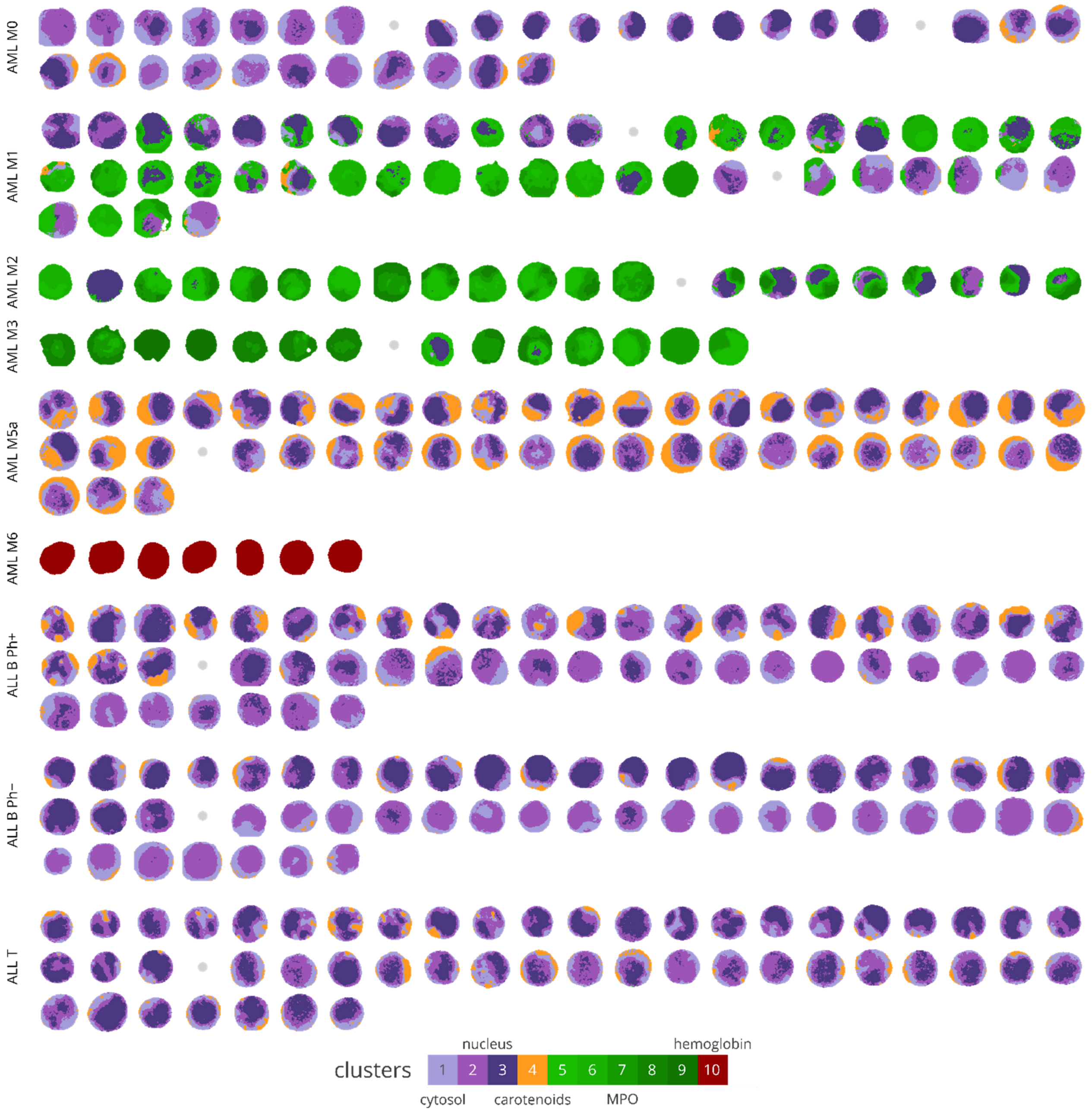

Figure S5 (10-level cluster analysis): *Pseudo-stained Raman images of 315 leukemia cells from 9 different leukemia subtypes, produced by a global whole-dataset cluster analysis limited to 10 clusters.*

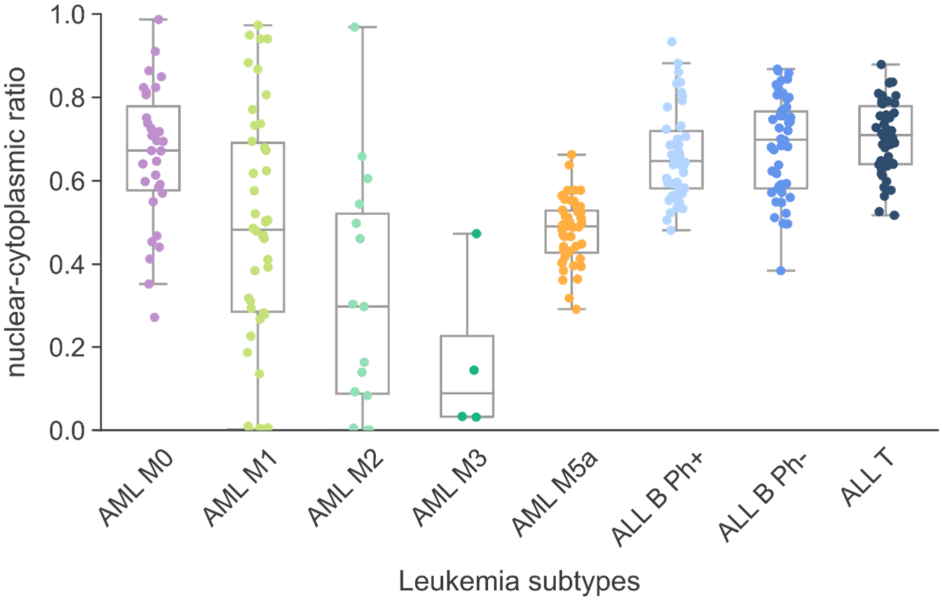

Figure S6 (nuclear-cytoplasmic ratios): *Box plot of the nuclear-cytoplasmic ratios for the different leukemia subtypes. For each cell, pixels belonging to the clusters #4–7 are considered as "nuclear" region, whereas the remaining pixels - those belonging to the cytoplasm (cluster #1–3), carotenoids (#8–9), MPO (#10–14), and hemoglobin (#15–17) clusters - are considered as the "cytoplasmic" region. AML M6 cells are not shown, because none of the pixels for this subtype were assigned to the "nucleus" clusters (4–7).*

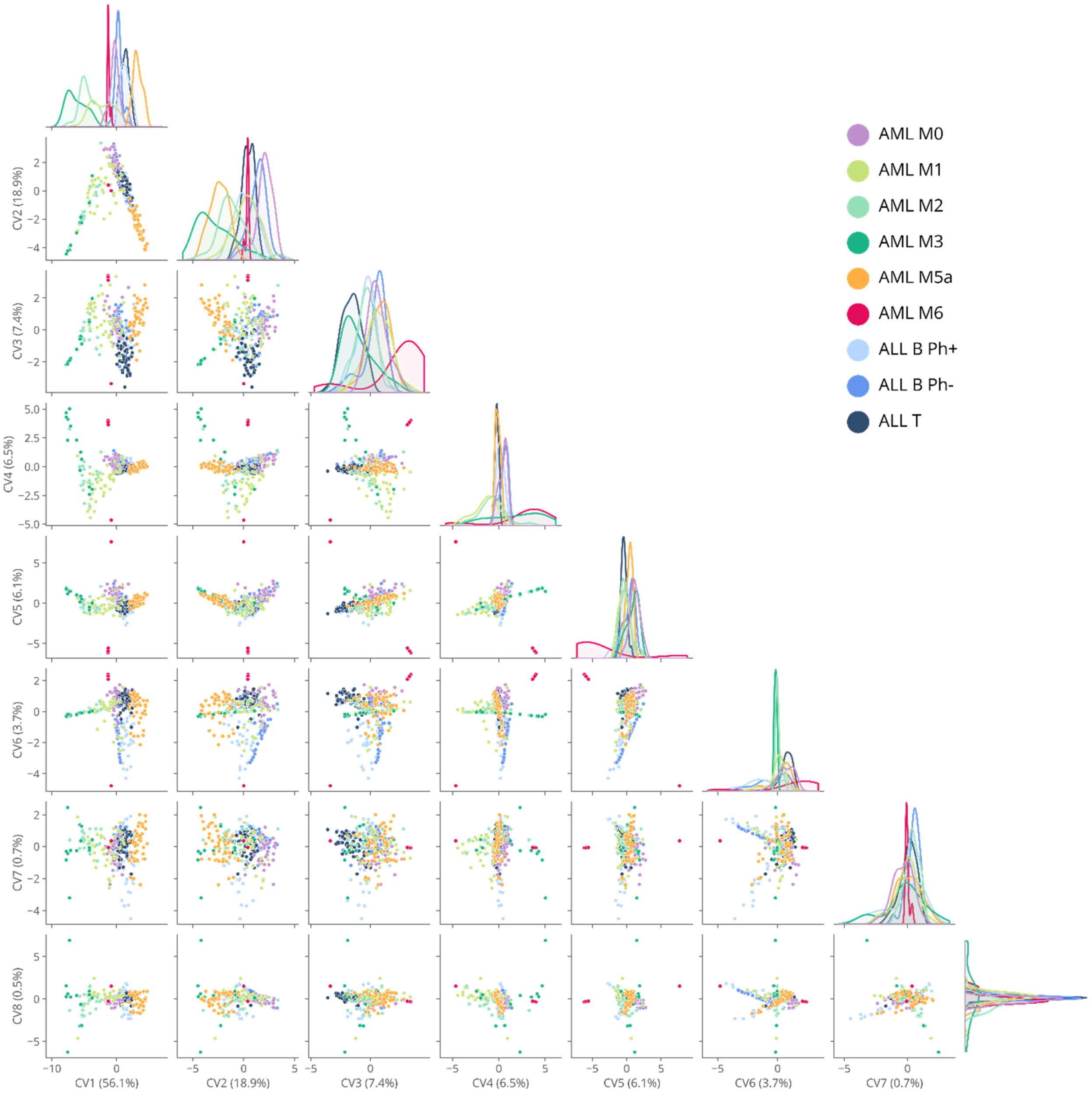

Figure S7 (LDA scatter plots for "AMLs+ALLs): *LDA scatter plots of the 8 CVs resulting from the LDA performed on the entire dataset ("AMLs+ALLs"). The kernel density estimate (KDEs) relative to AML M6 have been rescaled for visualization purposes. Percentage values in parentheses represent the proportions of variance explained by the corresponding CV.*

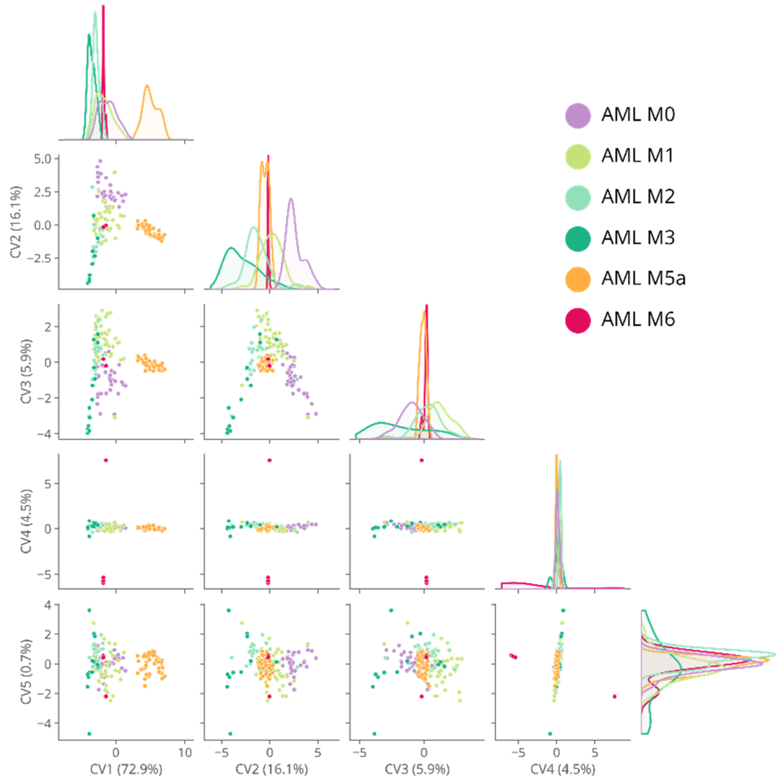

Figure S8 (LDA scatter plots for "AMLs"): *LDA scatter plots of the 5 CVs resulting from the LDA performed on AML subtypes ("AMLs" subset). The kernel density estimate (KDEs) relative to AML M6 have been rescaled for visualization purposes. Percentage values in parentheses represent the proportions of variance explained by the corresponding CV.*

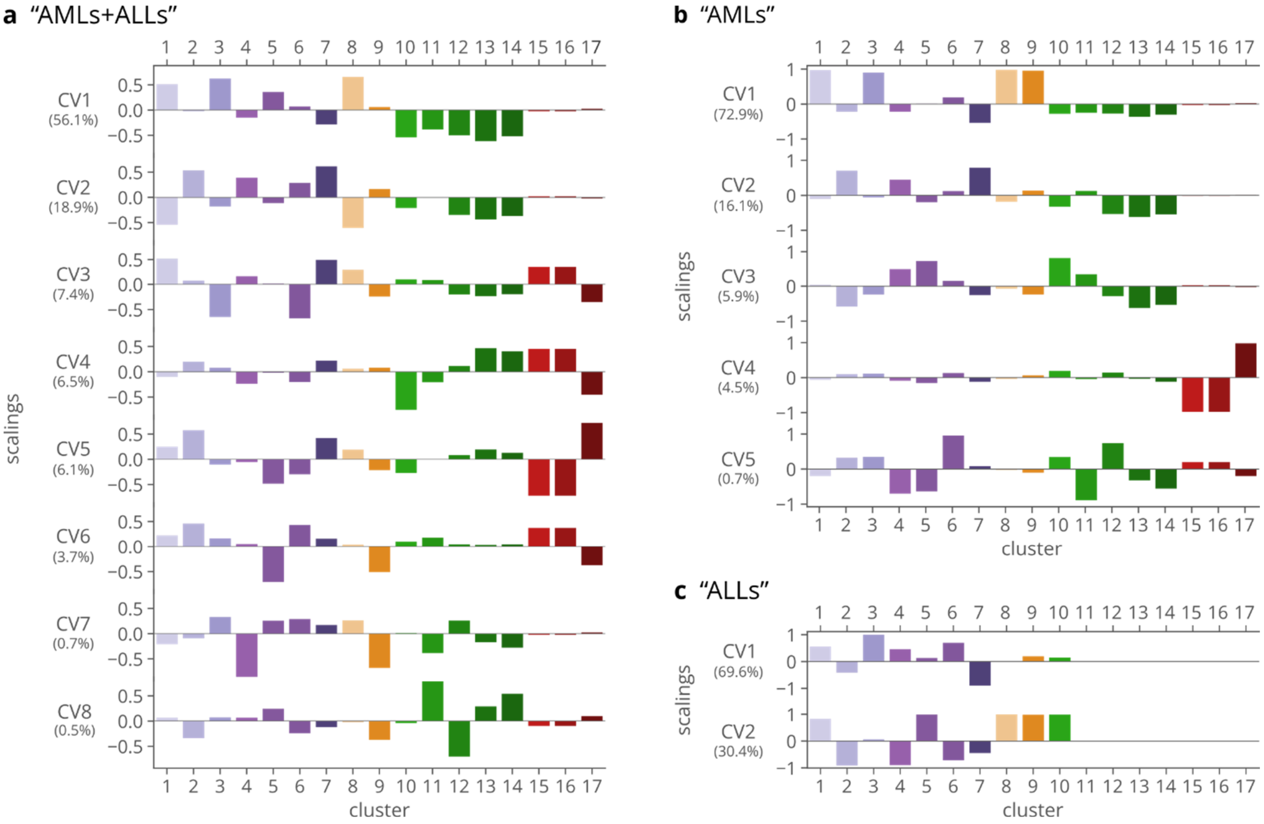

Figure S9 (LDA scalings): *Scalings relative to each canonical variable resulting from the LDA performed on, respectively,* ***a****) AML and ALL subtypes ("AMLs+ALLs"),* ***b****) AML subtypes ("AMLs"),* ***c****) ALL subtypes ("ALLs"). Percentage values in parentheses represent the proportion of variance explained by the corresponding CV.*

REFERENCES

(1) Vanna, R.; Ronchi, P.; Lenferink, A. T. M.; Tresoldi, C.; Morasso, C.; Mehn, D.; Bedoni, M.; Picciolini, S.; Terstappen, L. W. M. M.; Ciceri, F.; Otto, C.; Gramatica, F. Label-Free Imaging and Identification of Typical Cells of Acute Myeloid Leukaemia and Myelodysplastic Syndrome by Raman Microspectroscopy. *The Analyst* **2015**, *140* (4), 1054–1064. https://doi.org/10.1039/c4an02127d.

(2) Pully, V. V.; Lenferink, A. T. M.; Otto, C. Time-Lapse Raman Imaging of Single Live Lymphocytes. *J. Raman Spectrosc.* **2011**, *42* (2), 167–173. https://doi.org/10.1002/jrs.2683.
